# Trends, cross-country inequalities and risk factors in the global burden of chronic obstructive pulmonary disease mortality, 1990–2021: a secondary data analysis of the Global Burden of Disease Study 2021

**DOI:** 10.18332/tid/228906

**Published:** 2026-10-16

**Authors:** Yu Sun, Shu Li, Dan Xu, Yao Li, Wei Li

**Affiliations:** 1 Department of Cardiothoracic Surgery, The Affiliated Hospital of Jiaxing University, Jiaxing, China; 2 Department of Medical Administration, The Affiliated Hospital of Jiaxing University, Jiaxing, China; 3 Department of Blood Transfusion, The Affiliated Hospital of Jiaxing University, Jiaxing, China; 4 College of Rehabilitation and Health Care, Hunan Traditional Chinese Medical College, Zhuzhou, China; 5 Department of Clinical Laboratory, The Affiliated Hospital of Jiaxing University, Jiaxing, China

**Keywords:** chronic obstructive pulmonary disease, mortality, risk factors, cross-country inequality analysis, decomposition analysis

## Abstract

**INTRODUCTION:**

Chronic obstructive pulmonary disease (COPD) is the third leading cause of death worldwide. Understanding the mortality trends and influencing factors of chronic obstructive pulmonary disease is crucial for addressing systemic health disparities and informing fair decision-making.

**METHODS:**

This is a secondary data analysis based on the Global Burden of Disease Study 2021. The mortality burden of COPD from 1990 to 2021 was evaluated using decomposition analysis, risk factor analysis, health inequality analysis, and the autoregressive integrated moving average (ARIMA) prediction model.

**RESULTS:**

Globally, COPD deaths increased from 2495513.45 (95% UI: 2238986.25–2694755.05) in 1990 to 3719936.55 (95% UI: 3347912.32–4084218.2) in 2021, while the age-standardized mortality rate per 100000 population (ASMR) decreased from 71.92 (95% UI: 64.47–77.53) to 45.22 (95% UI: 40.61–49.7). Significant reductions in ASMR were observed in high and high-middle sociodemographic index (SDI) regions, whereas low and low-middle SDI regions saw minimal improvements. The highest ASMRs were noted in Oceania, South Asia, and East Asia. Risk factors such as environmental/occupational risks, air pollution, and tobacco use remained significant contributors to COPD mortality. Gender disparities were evident, with males having higher ASMRs than females, particularly after the age of 60 years. Cross-country health inequalities worsened, with the concentration index indicating a shift towards greater burden in low SDI countries. The future forecast indicated that ASMR will continue to decrease to 19.97 (95% CI: 10.98–28.96) by 2051.

**CONCLUSIONS:**

Despite overall improvements in COPD mortality rates, the increasing total number of deaths underscores the challenges posed by demographic shifts and health inequalities. Differentiated prevention and control strategies targeting low SDI regions, with attention to gender and older population differences, are needed to address key risk factors and reduce health inequalities.

## INTRODUCTION

COPD is a progressive chronic lung disease marked by persistent respiratory symptoms and impaired airflow in the airways^1^. Globally, COPD represents a significant public health challenge, accounting for approximately 3 million deaths annually, equivalent to 6% of all fatalities worldwide^2,3^. With a prevalence rate of up to 13.7% among individuals aged >40 years , COPD affects nearly 100 million people^4^. Projections suggest that the total number of COPD cases may rise to 600 million by 2050^5^. The growing burden of COPD is not only a health concern but also a reflection of global health inequalities. Disparities in healthcare access, environmental exposures, and socioeconomic conditions contribute to the differential impact of COPD across regions and populations. To develop effective prevention and treatment strategies, it is crucial for policymakers, healthcare professionals, and researchers to understand the mortality trends and associated risk factors of COPD at global, regional, and national levels.

The significance of this research is underscored by the complex interplay of risk factors contributing to COPD. These factors include smoking, environmental pollutants, occupational exposure, and tobacco, among others^6^. Environmental and occupational exposures, particularly air pollution (e.g. ambient particulate matter, household biomass fuels), are critical drivers of COPD mortality. These factors disproportionately affect low- and middle-income regions due to inadequate regulations and healthcare infrastructure, necessitating their inclusion in global burden analyses. Moreover, socioeconomic determinants and access to healthcare further complicate the landscape of COPD management and outcomes. Despite advances in medical knowledge and treatment options, the number of deaths from COPD continues to increase, highlighting the urgent need for comprehensive data analysis to inform targeted interventions. This study extends beyond merely quantifying disease burden; it seeks to unravel the structural drivers of health inequities, such as socioeconomic disparities, environmental injustice, and differential access to healthcare. These insights are vital for advocating global accountability in health governance and fostering collaborative strategies to mitigate preventable mortality.

This research was designed to analyze the trends in COPD mortality from 1990 to 2021 by leveraging data from the GBD Study 2021. By examining the variations in mortality rates both globally and within specific regions and nations, we aim to identify key risk factors and highlight populations at higher risk. Our findings underscore the necessity of transcending clinical frameworks to address COPD as a marker of broader social and environmental inequities, thereby advocating for policies that integrate health equity into sustainable development agendas. This analysis will shed light on the overarching patterns of COPD and contribute to evidence-based policy formulation aimed at reducing the burden of this preventable disease. Ultimately, through a thorough understanding of mortality trends and associated risk factors, we can advocate for enhanced public health initiatives and resource allocation dedicated to combating COPD on both national and international fronts.

## METHODS

### Data source

This is a secondary data analysis based on the Global Burden of Disease Study 2021 (GBD 2021). Estimates of the number of deaths and age-standardized mortality rate per 100000 population (ASMR) for COPD globally, regionally, and nationally from 1990 to 2021 were obtained from the GBD database. ASMR is the COPD mortality rate which is used to eliminate the influence of differences in the age structure of the population in different regions or years. COPD is characterized according to the criteria established by the Global Initiative for Chronic Obstructive Lung Disease^7^. The diagnostic criteria for COPD are defined by ICD-10. In addition to these mortality statistics, we gathered information on the proportion of COPD-related deaths that can be attributed to a variety of risk factors. These include, but are not limited to, air pollution in various forms, such as ambient ozone and particulate matter, along with behavioral risks, environmental and occupational hazards, elevated temperatures, household air pollution caused by the use of solid fuels, and tobacco use. Given that this study utilized publicly available data, there was no requirement for ethical approval to conduct the research.

### Statistical analysis

To acquire a thorough insight into the burden of COPD mortality, descriptive analyses were conducted at the global, 21 GBD geographical regions, and 204 country levels. In addition, in order to more systematically analyze the impact of the level of socioeconomic development on COPD mortality, we also included the SDI. The SDI serves as a composite measure to assess the extent of sociodemographic development in a nation or region, comprising five distinct areas. There were 204 countries or regions in the world, corresponding to 204 SDI values. Usually, the SDI is stratified from low to high in five categories: low (41), low-middle (41), middle (40), high-middle (41), and high (41). To compute the estimated annual percentage change (EAPC), a linear regression approach was employed^8^. The research examined trends related to the number of deaths caused by COPD and the ASMR from 1990 through 2021, along with the varying effects of gender and age on the ASMR associated with COPD.

To gain insight into the explanatory factors driving changes in the ASMR for COPD, this research employed decomposition analysis to control for variables and explore the impact of aging, population growth, and epidemiological changes on COPD mortality^9^. Health inequality analyses can serve as a basis for health planning and help to improve related policies and programs, thereby reducing disparities in the distribution of health. As previously described^10^, both the slope index of inequality and the concentration index were computed to evaluate disparities in the worldwide burden of COPD mortality. An age-period-cohort model was used to account for the effects of age, period, and cohort on COPD mortality. The ARIMA model was specifically utilized to forecast trends in COPD mortality rates over the forthcoming three decades^11^. This statistical approach enables researchers to analyze historical data and project future outcomes, making it a valuable tool in public health planning. The confidence interval for each variable was 95% CI. All statistical analyses were conducted utilizing R software (version 4.3.1). A significance level of p<0.05 was deemed statistically significant.

## RESULTS

### The global, regional, and national COPD mortality

In 1990, there were an estimated 2495513.45 (95% UI: 2238986.25–2694755.05) deaths from COPD worldwide, with an ASMR of 71.92 (95% UI: 64.47–77.53). By 2021, COPD-related deaths surged to 3719936.55 (95% UI: 3347912.32–4084218.2) globally, but the ASMR had decreased to 45.22 (95% UI: 40.61–49.7) (Table 1). This divergence suggests that, despite the increasing number of deaths, improvements in population-level health-potentially attributable to enhanced disease management, preventive measures, or changes in demographic composition-may have contributed to a reduced per capita mortality risk. The high SDI region has the lowest ASMR of 25.53 (95% UI: 23.71–26.5) and 19.44 (95% UI: 17.26–20.66) in 1990 and 2021 respectively, with the total number of deaths increasing from 287971.17 (95% UI: 270268.54–300481.59) to 469238.51 (95% UI: 410389.19–501052.68) during the same period (Table 1). In 1990, the ASMR in the high-middle SDI region was 79.53 (95% UI: 70.61–86.61). By 2021, however, its ASMR declined markedly to 35.91 (95% UI: 30.78–40.69), corresponding to an EAPC of -3.15 (95% CI: -3.39 − -2.91), the steepest decline among the five SDI regions (Table 1). In 1990, the ASMR in the middle SDI region was 123.89 (95% UI: 109.19–134.8), which was significantly higher than the other regions. However, by 2021, the ASMR decreased to 57.45 (95% CI: 49.59–65.43) and the EAPC was -2.85 (95% CI: -2.98 – -2.72) (Table 1). Between 1990 and 2021, high-middle and middle SDI regions achieved notable reductions in COPD mortality, reflecting advancements in disease management. Conversely, low-middle and low SDI regions exhibited persistently elevated ASMR by 2021. For example, while the ASMR in low-middle SDI regions declined from 92.07 (95% UI: 74.17–107.46) to 84.76 (95% UI: 75.8–93.78) during this period, total COPD deaths nearly doubled from 443218.09 (95% CI: 352861.5–517320.13) to 999762.37 (95% CI: 898211.09–1104472.17), accompanied by an EAPC of -0.12 (95% CI: -0.23 – -0.02) (Table 1). Similarly, low SDI regions experienced only a modest ASMR decrease from 77.67 (95% UI: 61.91–91.44) to 70.7 (95% CI: 63.35–79.76), despite a sharp rise in fatalities from 133099.95 (95% CI: 106538.23–156186.18) to 266003.52 (95% CI: 237374.65–300954.63) and an EAPC of -0.11 (95% UI: -0.27–0.05) (Table 1). These stagnating trends underscore persistent systemic health challenges, including inequitable access to care and insufficient public health infrastructure, in lower resource settings.

**Table 1:** Mortality of COPD Between 1990 and 2021 at the global and regional level

*Location*	*1990* *Deaths (95% UI)*	*1990* *ASMR (95% UI)*	*2021* *Deaths (95% UI)*	*2021* *ASMR (95% UI)*	*EAPC* *(95% CI)*
**Global**	2495513.45 (2238986.25–2694755.05)	71.92 (64.47–77.53)	3719936.55 (3347912.32–4084218.2)	45.22 (40.61–49.7)	-1.75 (-1.84 – -1.65)
**SDI regions**					
High	289771.17 (270268.54–300481.59)	25.53 (23.71–26.5)	469238.51 (410389.19–501052.68)	19.44 (17.26–20.66)	-0.97 (-1.04 – -0.9)
High-middle	673578.23 (599623.03–733445.51)	79.53 (70.61–86.61)	689449 (591990.84–781694.4)	35.91 (30.78–40.69)	-3.15 (-3.39 – -2.91)
Middle	954485.27 (837573.01–1044246.29)	123.89 (109.19–134.8)	1293660.1 (1122165.5–1476736.57)	57.45 (49.59–65.43)	-2.85 (-2.98 – -2.72)
Low-middle	443218.09 (352861.5–517320.13)	92.07 (74.17–107.46)	999762.37 (898211.09–1104472.17)	84.76 (75.8–93.78)	-0.12 (-0.23 – -0.02)
Low	133099.95 (106538.23–156186.18)	77.67 (61.91–91.44)	266003.52 (237374.65–300954.63)	70.7 (63.35–79.76)	-0.11 (-0.27 – 0.05)
**Regions**					
Andean Latin America	3371.51 (2925.64–3779.77)	19.44 (16.88–21.8)	7390.43 (6022.51–8969.89)	13.4 (10.93–16.29)	-0.88 (-0.98 – -0.77)
Australasia	6957.83 (6537.55–7311.96)	29.42 (27.56–30.92)	11514.97 (10072.77–12490.73)	18.89 (16.66–20.42)	-1.57 (-1.78 – -1.36)
Caribbean	4363.15 (3750.19–4857.67)	18.48 (16.1–20.5)	10847.4 (9545.67–12273.91)	19.85 (17.47–22.49)	0.14 (0–0.29)
Central Asia	15214.76 (14273.11–16065.14)	36.33 (34.02–38.45)	14472.3 (12972.34–16004.45)	21.27 (19.12–23.46)	-2.09 (-2.4 – -1.79)
Central Europe	42641.79 (41049.28–44041.07)	30.65 (29.4–31.73)	37944.37 (34726.9–40751.7)	16.03 (14.7–17.22)	-1.89 (-2.06 – -1.73)
Central Latin America	23530.52 (22316.36–24216.18)	35.73 (33.58–36.9)	62380.92 (54936.77–68713.47)	26.92 (23.7–29.64)	-1.03 (-1.15 – -0.92)
Central Sub-Saharan Africa	8212.88 (5712.68–11059.28)	52.33 (36.88–72.56)	16404.54 (11384.81–24013.97)	42.82 (29.63–65.02)	-0.73 (-0.77 – -0.69)
East Asia	1258158.14 (1083429.31–1405339.51)	225.27 (194.01–250.33)	1323440.99 (1082507.99–1574211.39)	72.2 (59.32–85.26)	-4.2 (-4.42 – -3.98)
Eastern Europe	92296.05 (88866–94716.94)	35.77 (34.15–36.74)	42153.21 (38785.56–45390.03)	11.72 (10.79–12.62)	-4.43 (-4.77 – -4.08)
Eastern Sub-Saharan Africa	24211.07 (18456.4–27823.28)	42.29 (32.1–48.71)	38601 (31589.67–45499.16)	29.91 (24.41–35.14)	-1.31 (-1.38 – -1.24)
High-income Asia Pacific	24945.76 (22675.29–26379.09)	14.17 (12.76–15.08)	44250.88 (36742.22–49170.48)	6.68 (5.7–7.37)	-2.53 (-2.74 – -0.33)
High-income North America	97862.48 (90388.63–101601.43)	26.28 (24.3–27.29)	213210.91 (185696.61–226184.97)	29.89 (26.2–31.63)	0.35 (0.15–0.55)
North Africa and Middle East	47247.5 (37557.17–53481.62)	36.17 (28.39–41.23)	92679.97 (82194.3–103229.07)	26.37 (23.19–29.31)	-0.87 (-1.03 – -0.72)
Oceania	2947.15 (2265.57–3751.53)	144.27 (113.03–180.83)	6218.94 (4984.92–7671.74)	118.21 (96.32–144.59)	-0.7 (-0.73 – -0.67)
South Asia	500887.44 (386528.44–592094.06)	114.54 (89.16–136.08)	1229609.51 (1104444.79–1381283.99)	101.63 (90.55–114.34)	-0.22 (-0.36 – -0.08)
Southeast Asia	119331.72 (96930.6–135191.54)	60.18 (48.55–68.27)	228487.39 (202410.2–257524.96)	43.14 (38.24–48.54)	-1.18 (-1.3 – -1.06)
Southern Latin America	11505.19 (10858.78–12127.22)	26.92 (25.28–28.4)	20618.94 (18764.22–22039.45)	22.53 (20.53–24.07)	-0.43 (-0.71 – -0.14)
Southern Sub-Saharan Africa	8397.39 (7473.51–9962.22)	37.4 (32.85–44.78)	16579.1 (15267.62–17780.86)	34.78 (31.93–37.34)	-0.39 (-0.75 – -0.03)
Tropical Latin America	32678.63 (30713.6–33995.11)	44.64 (41.13–46.88)	64662.04 (57768.74–68635.22)	26.32 (23.44–27.99)	-2.1 (-2.35 – -1.85)
Western Europe	150667.65 (140414.01–156295.97)	24.66 (22.93–25.6)	205393.48 (177939.48–219861.62)	17.82 (15.74–18.92)	-1 (-1.08 – -0.92)
Western Sub-Saharan Africa	20084.82 (15852.53–23874.93)	28.53 (22.44–33.71)	33075.26 (29016.9–37675.25)	22.07 (19.45–25.05)	-0.65 (-0.74 – -0.56)

ASMR: age-standardized mortality rate per 100000 population. EAPC: estimated annual percentage change. UI: uncertainty interval. CI: confidence interval.

ASMR for COPD varies widely among regions around the world, with Oceania observing the highest ASMR at 118.21 (95% CI: 96.32–144.59) in 2021, followed by South Asia and East Asia (Figure 1). As for the absolute numbers, East Asia and South Asia recorded more than two-thirds of the deaths in 2021 (1323440.99; 95% UI: 1082507.99–1574211.39, and 1229609.51; 95% UI: 1104444.79–1381283.99, respectively), followed by Southeast Asia and high-income North America. Eastern Europe is the region with the highest decline in ASMR, from 35.77 (95% UI: 34.15–36.74) in 1990 to 11.72 (95% UI: 10.79–12.62) in 2021, with an EAPC of -4.43 (95% CI: -4.77 – -4.08). In 1990, East Asia had an ASMR of 225.27 (95% UI: 194.01–250.33), significantly higher than other regions. By 2021, the ASMR in East Asia was reduced to 72.2, and the EAPC was -4.2 (95% CI: -4.42 – -3.98). However, the total number of deaths increased from 1258158.14 (95% UI: 1083429.31–1405339.51) to 1323440.99 (95% UI: 1082507.99–1574211.39). South Asia reported an ASMR of 114.54 (95% CI: 89.16–136.08) and 101.63 (95% CI: 90.55–114.34) for 1990 and 2021, respectively, and an EAPC of -0.22 (95% UI: -0.36 – -0.08). Despite improvements in the region, the total number of deaths increased dramatically from 500887.44 (95% CI: 386528.44–592094.06) to 1229609.51 (95% CI: 1104444.79–1381283.99), reflecting continuing public health challenges. The Oceania region had an ASMR of 144.27 (95% CI: 113.03–180.83) in 1990, reduced to 118.21 (95% CI: 96.32–144.59) in 2021, and an EAPC of -0.7 (95% UI: -0.73 – -0.67). While its ASMR remains high, its total number of deaths is relatively low, increasing from 2947.15 (95% CI: 2265.57–3751.53) deaths in 1990 to 6218.94 (95% CI: 4984.92–7671.74) in 2021. Supplementary file Figure S1 illustrates the observed regional and national ASMR in relation to the SDI, compared with the expected levels for each location based on SDI. High-income regions, as well as central and eastern Europe, and tropical and southern Latin America, generally aligned with the expected trends throughout the study period. In contrast, substantial variation was observed across many middle SDI regions. A positive correlation between ASMR and SDI was evident when SDI was below 0.45, whereas an inverse correlation was observed when SDI exceeded 0.45.

**Figure 1:**
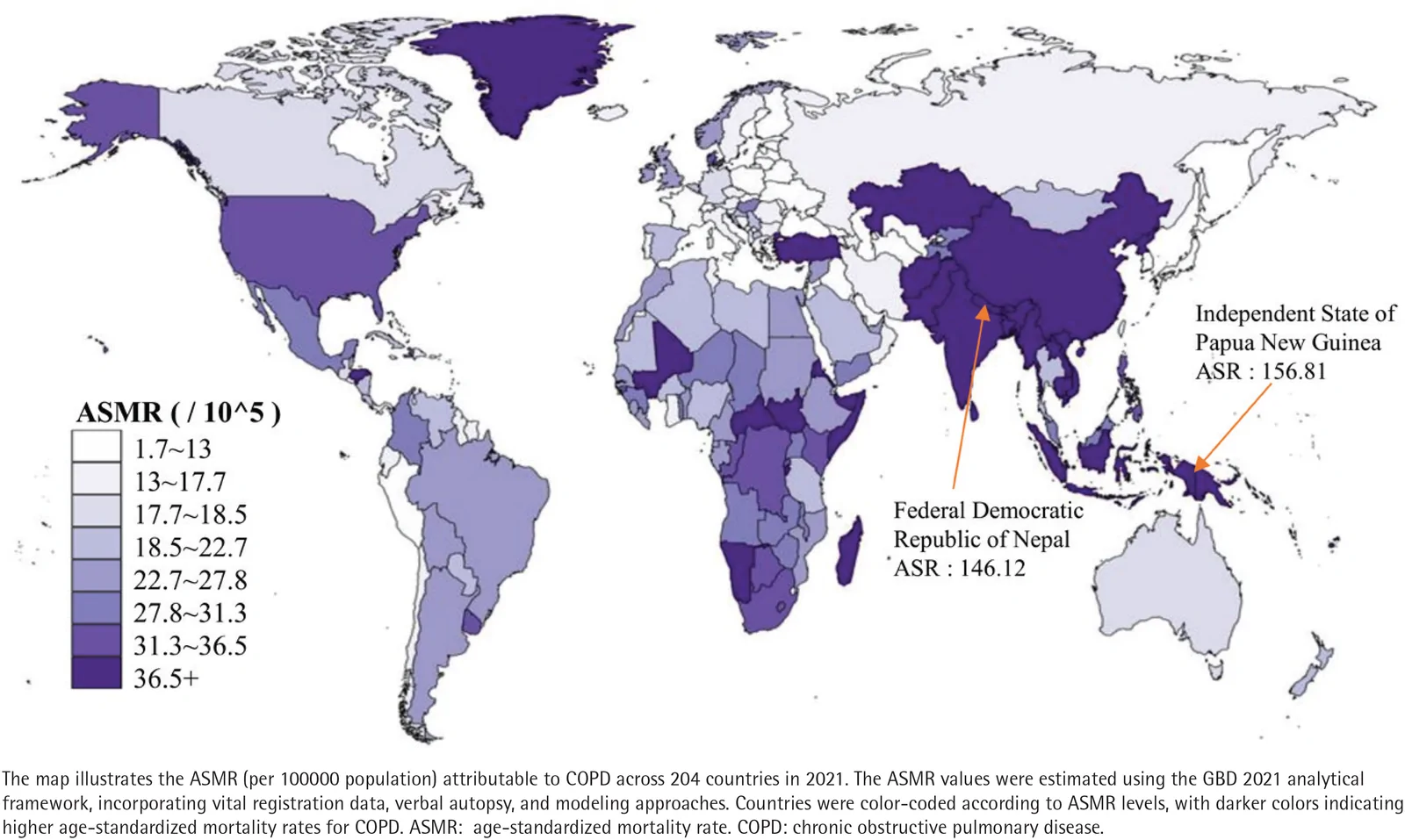
Global ASMR of COPD in 204 countries and territories, 2021

Of the 204 nations examined, the one that recorded the highest ASMR in 2021 is the Independent State of Papua New Guinea, which has an ASMR of 156.81 (95% CI: 123.55–197.43). This is followed by the Federal Democratic Republic of Nepal, the Republic of India, and the Democratic People’s Republic of Korea (Figure 1; and Supplementary file Figure S2). Belize (347.87%) had the most significant increase in mortality, followed by Venezuela (297.08%) and Honduras (281.16%). In contrast, Belarus (-79.82%) had the most significant mortality reduction, followed by Ukraine (-77.40%) (Figure 2). Norway exhibited the most pronounced rise in ASMR, with an EAPC of 2.14, followed by Grenadines at 1.56. Conversely, Singapore demonstrated the steepest ASMR decline (EAPC= -5.63), trailed by Belarus (EAPC= -5.55) and Ukraine (EAPC= -4.94) (Figure 3; and Supplementary file Figure S2).

**Figure 2:**
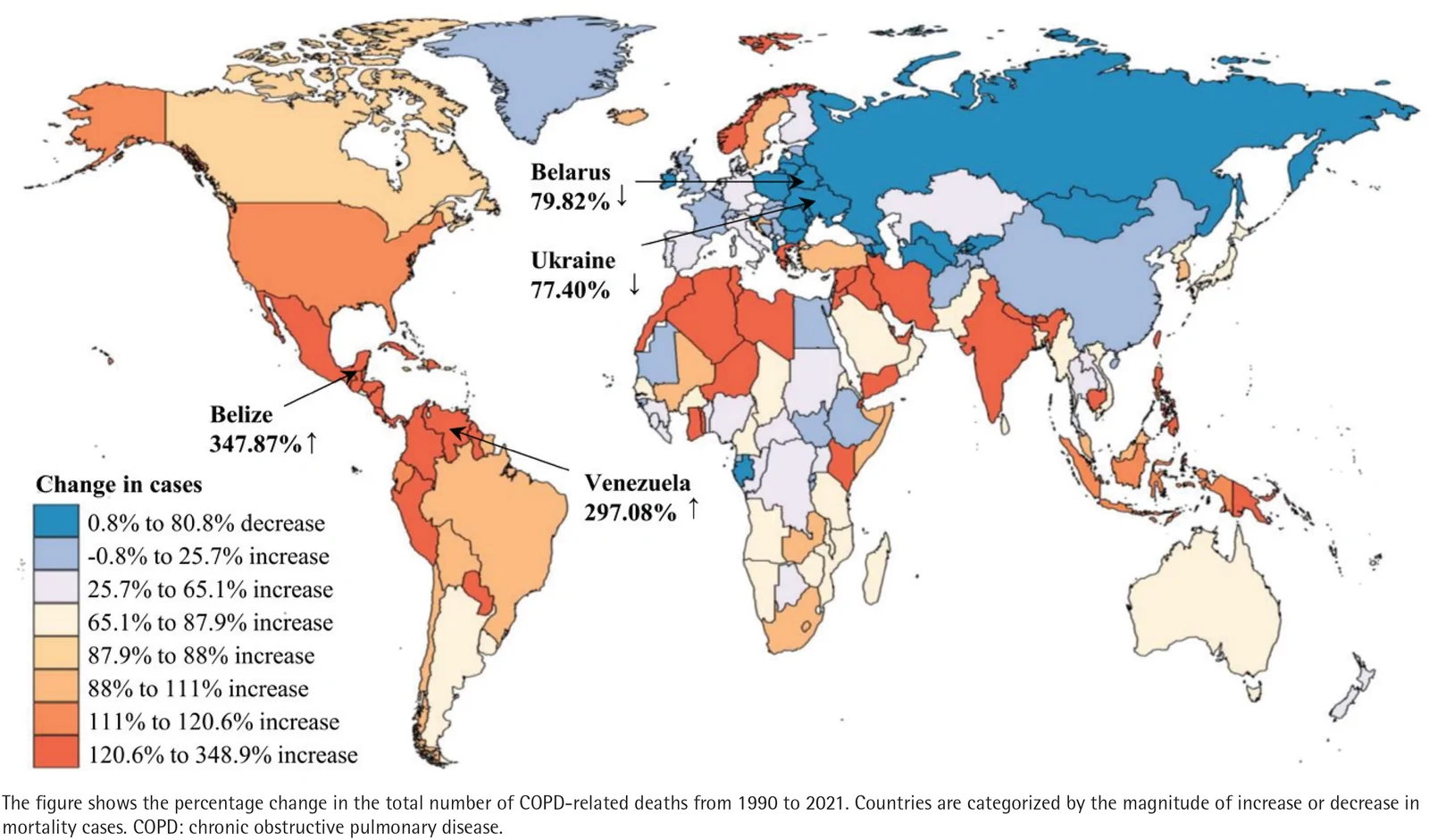
Relative change in COPD mortality cases between 1990 and 2021 across 204 countries

**Figure 3:**
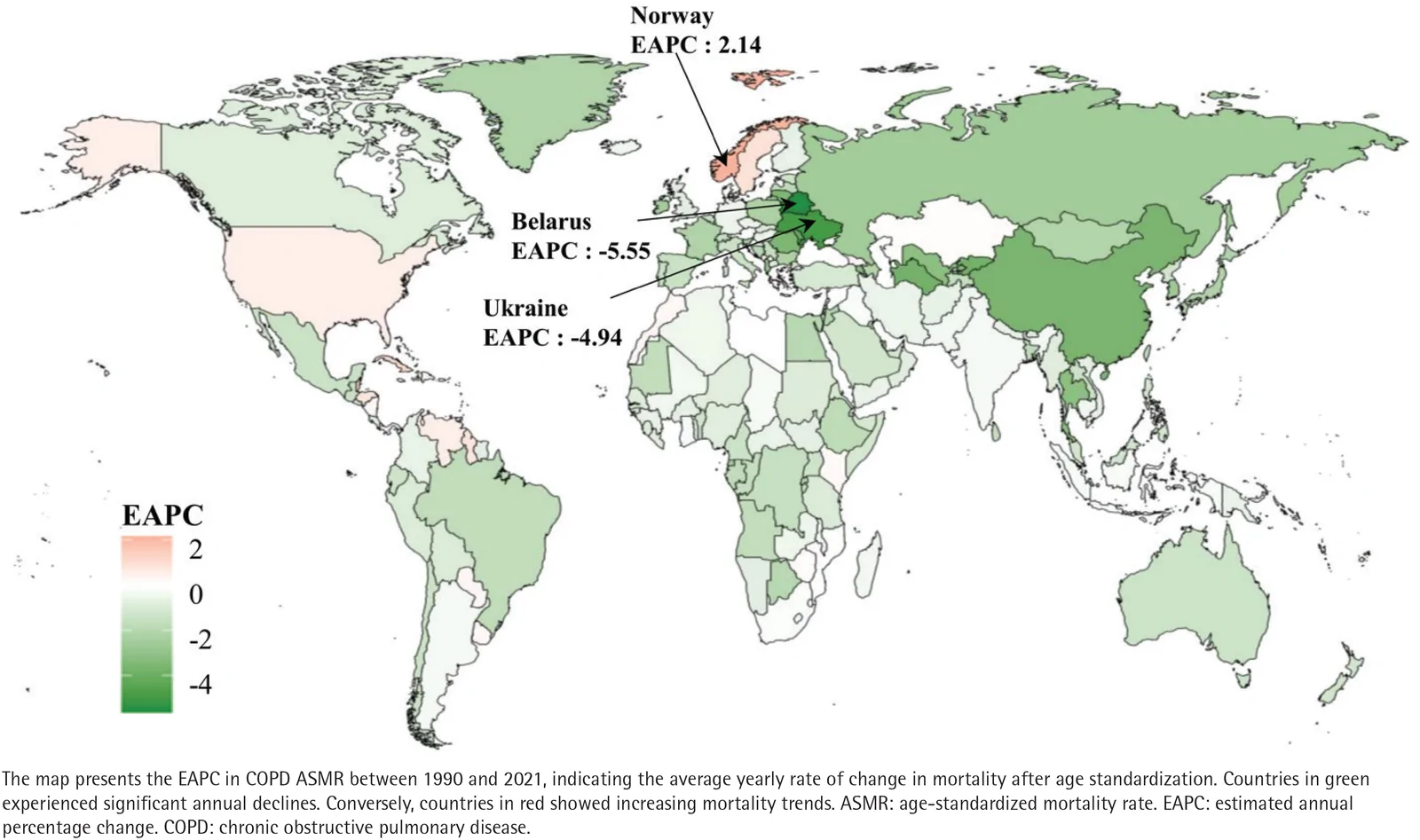
EAPC in ASMR for COPD from 1990 to 2021 across 204 countries

### Effects of age and gender

For all genders, global ASMR showed a steady decline from 1990 to 2021, with a larger decline for men than for women. Among the five SDI regions, the middle SDI region demonstrated the most notable reduction in ASMR, followed closely by the high-middle SDI region. The high SDI region maintained the lowest mortality burden with only a modest downward trend. Low-middle and low SDI regions exhibited minimal overall progress despite fluctuating ASMR values, persistently retaining the highest mortality rates (Supplementary file Figure S3A). Gender-stratified analysis revealed parallel trends in ASMR for males and females compared to the combined sex-specific rates. However, males generally showed a higher ASMR than females (Supplementary file Figure S3A). A breakdown of global COPD mortality rates by age group and sex reveals that high COPD mortality rates (92.90%) are concentrated in those aged >60 years, with a smaller impact on those aged <60 years (Supplementary file Figure S3B). It can be observed that within almost all age groups, mortality rates are significantly higher in men than in women, a trend that is particularly pronounced after the age of 60 years and progressively increases with age (Supplementary file Figure S3B). In summary, ASMR in COPD is significantly influenced by age and gender, and there are significant differences between regions with different SDI stratification.

### Cross-country inequality analysis

Between 1990 and 2021, the slope index of inequality rose from 10.5 (95 % CI: 4.99–16.0) to 18.39 (95 % CI: 12.90–23.89) (Supplementary file Figure S4A). Furthermore, the concentration index was -0.11 (95 % CI: -0.16 – -0.05) in 1990 and shifted to -0.15 (95 % CI: -0.21 – -0.09) in 2021. Although the concentration index showed minimal change, the curve exhibited greater volatility in 2021, indicating a transformation in the nature of inequality (Supplementary file Figure S4B). Notably, low-income countries disproportionately contributed to global COPD mortality by 2021. Collectively, these findings underscore a progressive escalation in health inequities related to COPD across the three-decade study period.

### Decomposition analysis of ASMR

Using decomposition analysis, this study assessed the relative contributions of aging, population growth, and epidemiological changes to COPD mortality trends between 1990 and 2021. Globally, the effect of epidemiological changes demonstrated a consistent decline across all SDI regions, with the most pronounced reduction observed in middle SDI regions (Supplementary file Figure S5A). Conversely, population growth exerted an increasing influence on mortality rates worldwide, particularly in middle and low-middle SDI regions, where its contribution was most substantial. Similarly, aging populations drove rising mortality burdens in all SDI categories, with the greatest effect again concentrated in middle and low-middle SDI regions (Supplementary file Figure S5A). The overall difference in the epidemiological changes of COPD between male and female groups was not significant, and the effects of population growth and aging in men were more significant than those in women (Supplementary file Figure S5B). These findings highlight the divergent roles of demographic and epidemiological drivers in shaping COPD mortality trends over time.

### Risk factors for COPD mortality

From 1990 to 2021, the global trend showed a general decline in the contributions of each risk factor to COPD mortality (Supplementary file Figure S6A). Environmental/occupational risks (26.97%), air pollution (21.51%), particulate matter pollution (18.5%), behavioral risks (18.26%), tobacco (18.26%), and smoking (16.12%) remained the most important factors that influenced global COPD mortality in 2021 (Supplementary file Figures S6A and S6B). The impact of various risk factors on the mortality rates associated with COPD exhibits notable variations across different sociodemographic categories (Supplementary file Figure S6A). COPD mortality was lower in the high SDI regions and was less influenced by different risk factors. The COPD mortality rate in regions with high SDI saw a notable decline between 1990 and 2021. During this period, significant risk factors included behavioral risks (7.80%), tobacco use (7.80%), and smoking (7.43%). Conversely, in the high-middle SDI area, the influence of each risk factor on COPD mortality significantly diminished from 1990 to 2021, although environmental and occupational hazards (18.4%), air pollution (12.6%), tobacco (16.5%), smoking (14.7%), and behavioral risks (16.5%) continued to play crucial roles as risk factors. In the middle SDI regions, where COPD mortality decreased significantly from 1990 to 2021, environmental/occupational risks (33.67%), air pollution (25.83%), tobacco (24.19%), and behavioral risks (24.19%) were significant risk factors, all of which improved but remained prominent. Mortality rates in low-middle SDI regions and low SDI regions did not significantly decrease and remained very high. Environmental/occupational risks (63.20% and 57.37%, respectively), air pollution (57.60% and 53.7%, respectively), particulate matter pollution (51.40% and 49.8%, respectively), and household air pollution from solid fuels (33.31% and 39.1%, respectively) were the most important factors affecting COPD mortality in the low-middle SDI region and low SDI region (Supplementary file Figure S6B).

Risk factor contributions to COPD mortality exhibited marked regional heterogeneity (Supplementary file Figure S7). In Africa, environmental/occupational hazards (18.6%) and air pollution-related exposures-including ambient air pollution (16.3%), particulate matter (15.0%), and household air pollution from solid fuel combustion (10.3%) – collectively dominated the mortality burden, accounting for 60.2% of attributable deaths. In addition, these four risk factors are also major factors affecting mortality in Western, Central and Eastern Sub-Saharan Africa. Environmental/occupational risks (77.2%), air pollution (71.1%) and particulate matter pollution (62.5%) explain the higher mortality in South Asia. For Oceania, environmental/occupational risks (82.2%), air pollution (76.0%), particulate matter pollution (75.8%) and household air pollution from solid fuels (66.9%) were the most important causes affecting COPD mortality. For the Asian region, mortality was influenced by a variety of factors, including environmental/occupational risks (42.1%), behavioral risks (27.1%), air pollution (34.9%), tobacco (27.1%), particulate matter pollution (30.5%), ambient particulate matter pollution (16.9%) and smoking (23.7%).

### Age-period-cohort effect on age-standardized mortality rates of COPD

Supplementary file Figure S8 illustrates the effect of age-period-cohort on COPD mortality. Within the same birth cohort, mortality increased progressively with increasing age (Supplementary file Figure S8A). In the period rate ratio (RR) results of the current study, the mortality rate decreased slowly compared with that in recent years (Supplementary file Figure S8B). The risk of death from COPD was higher among people born earlier (1900–1940). With the advancing of birth year, the rate ratio value gradually decreased. The risk was significantly lower among people born later (after 1940) (Supplementary file Figure S8C).

### Prediction of COPD mortality in the next three decades

The ARIMA model was used to predict trends in COPD mortality over the next three decades. As shown in Figure 4, it is evident that the actual ASMR for COPD started at a relatively high level in 1990, about 71.92 (95% UI: 64.47–77.53), and gradually declined over the subsequent decades. There are slight fluctuations observed in the actual mortality rates from the late 1990s to about 2010. In 2021, the actual ASMR for COPD has decreased to 45.22 (95% UI: 40.61–49.7). The forecasted trajectory suggests a continued decrease, with ASMR projected to reach 37.81 (95% UI: 33.00–42.63) in 2030, 29.32 (95% UI: 22.20–36.43) in 2040, and 20.82 (95% UI: 11.98–29.66) in 2050. The yellow projection line indicates that the ASMR for COPD will continue to decline by 2051 and is projected to approach 19.97 (95% UI: 10.98–28.96).

**Figure 4:**
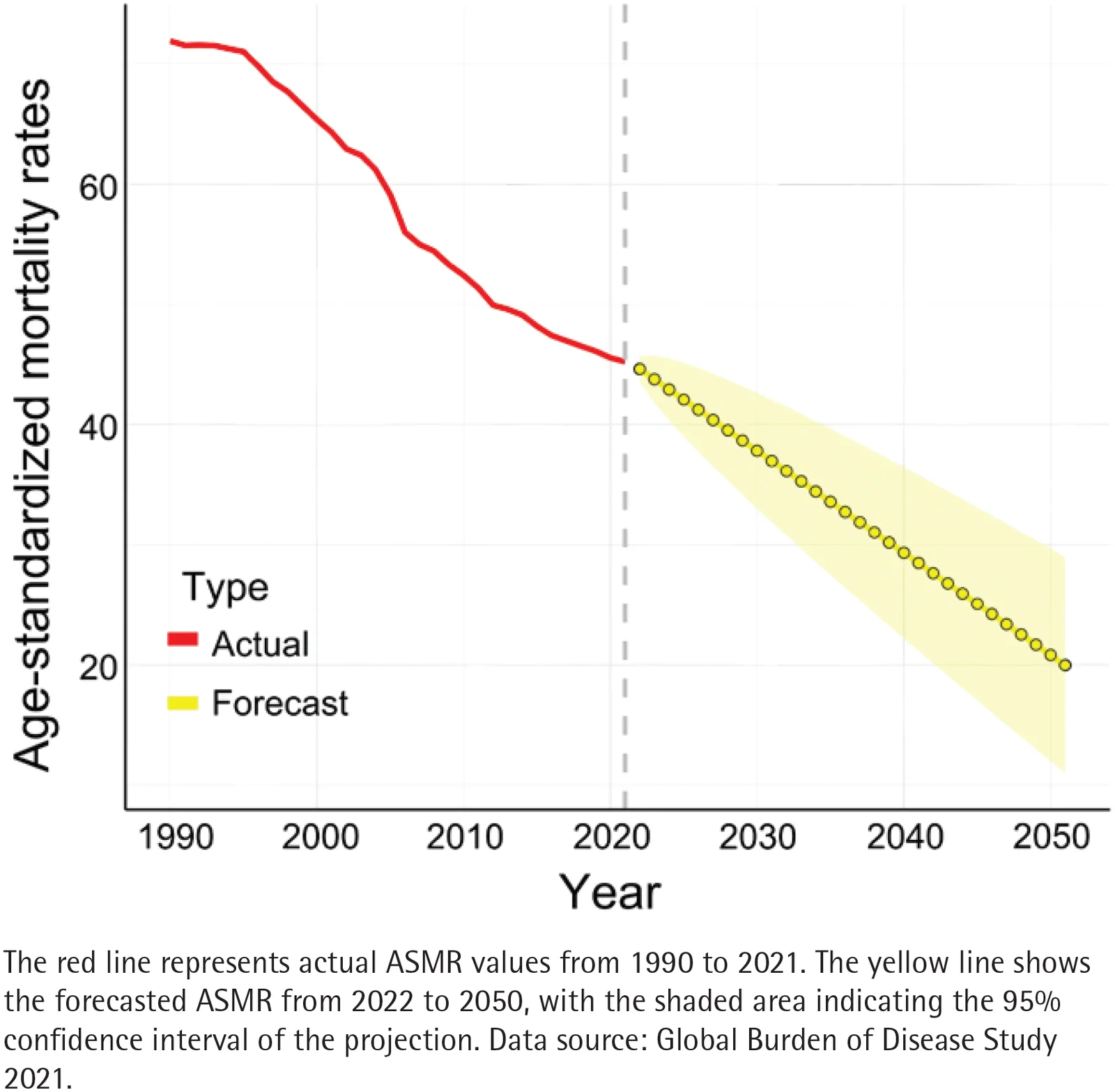
Prediction of trends in COPD mortality over the next three decades

## DISCUSSION

COPD ranks as the third most common cause of mortality worldwide; combined with the intricacies of its underlying mechanisms, it presents a major challenge in international public health^12^. We acknowledge that several studies have investigated the global burden of COPD^13-16^. These studies have made significant contributions to our understanding of COPD epidemiology. However, our study provides several novel insights and advancements. By utilizing the latest data from the GBD study, this research reveals the differences in COPD mortality rates across various SDI regions, age groups, genders, and countries. In addition, this study investigates the risk factors that contribute to COPD mortality and analyzes future trends in COPD mortality. This research not only improves our comprehension of the worldwide impact of COPD but also offers a scientific foundation for developing specific prevention and control measures in the future.

### Global trends: coexistence of progress and challenges

On the surface, the ASMR for COPD globally has decreased by 37.12% over the past 31 years (from 71.92 to 45.22), seemingly a significant success of public health interventions. Nevertheless, the overall count of fatalities has paradoxically risen by 49% (from 2.496 million to 3.719 million), revealing the profound impact of demographic changes. Taking East Asia as an example, Japan achieved a 49.18% reduction in the ASMR for COPD from 1990 to 2021 through the implementation of stringent smoking restrictions and effective control of 2.5 μm particulate matter (PM2.5)^17^. However, a notable rise in the percentage of the senior demographic is expected to lead to an elevated mortality rate among those aged ≥80 years. Similarly, in Sub-Saharan African countries, although ASMR has decreased, rapid population growth has resulted in a doubling of total deaths. These data reveal the complex interplay between population aging and the effectiveness of disease control, particularly in regions experiencing rapid aging or population growth.

A particularly troubling trend is the worsening of global health disparities. The concentration index, which serves as a measure of health inequality, has seen a decline from -0.11 in 1990 to -0.15 in 2021. This downward movement signifies an alarming shift in the distribution of COPD mortality, highlighting that the burden is increasingly being borne by countries with low SDI. This indicates that the gap between wealthier nations and those with fewer resources is widening, leading to significant public health concerns. For instance, the ASMR in Papua New Guinea reached 156.81 in 2021, approximately ten times that of Iceland, which recorded an ASMR of 16.86, despite the significant disparity in per capita healthcare expenditure between the two countries. This inequality arises not only from a scarcity of medical resources but is also deeply rooted in differences in environmental exposure. Residents of low SDI countries face dual threats from indoor biomass fuel use – where approximately 80% of households in Sub-Saharan Africa depend on wood combustion – and outdoor PM2.5 concentrations^18^, which exceed an annual average of 75 μg/m^3^ in several South Asian nations. Conversely, high SDI countries have substantially reduced exposure levels through energy transitions, exemplified by the European Union’s target of achieving a 45% share of renewable energy by 2030, alongside stringent controls on industrial pollution^19^. Moreover, high SDI regions often engage in the practice of industrial transfer, outsourcing pollution-intensive industries to lower income countries, thereby shifting health risks. This phenomenon of ‘environmental colonialism’ is particularly pronounced in the market expansion strategies of multinational tobacco companies. While this study underscores significant cross-country disparities in COPD mortality, it is important to acknowledge that within-country inequalities were not addressed. Subnational variations in the burden of COPD – driven by differences in socioeconomic status, geographical location, ethnicity, and access to healthcare – may be considerable, particularly in large and demographically diverse countries such as China, India, Brazil, and the United States. For instance, rural populations and marginalized communities are often disproportionately exposed to risk factors such as biomass fuel use and occupational hazards, while simultaneously experiencing limited access to diagnostic and therapeutic services. The GBD 2021 study provides subnational estimates for selected countries, and future research should utilize these data to examine trends in within-country inequities and support the development of more equitable health policy interventions.

### A dynamic game of risk factors: from traditional to emerging threats

Smoking remains a significant contributing factor to COPD mortality, but its impact displays considerable regional heterogeneity. In high SDI regions, measures such as tobacco control legislation, consumption tax reforms, and regulations on e-cigarettes (like the FDA’s prohibition of flavored e-cigarette sales in the United States) have resulted in significant reductions in smoking-related deaths^20^. Conversely, in low SDI countries, a lack of regulation and industry lobbying have resulted in an increase in tobacco consumption, exemplified by a male smoking rate as high as 76% in Indonesia^21^. It is worth noting that female exposure patterns present unique risk characteristics. In Sub-Saharan Africa and South-East Asia, the population attributable fraction for females exposed to biomass fuels was 28.2% and 17.8%, respectively^22^. Their pathological phenotype is characterized mainly by small airway lesions, with a higher frequency of acute exacerbations compared to men. However, globally, few COPD clinical trials have included gender-specific analyses, highlighting gender blindness in the research paradigm.

The pathogenic mechanisms of ambient particulate matter are being reconceptualized with advances in toxicology. PM2.5 can breach the alveolar–capillary barrier and access the bloodstream, which in turn speeds up the advancement of emphysema by causing damage to mitochondrial DNA and triggering the activation of NLRP3 inflammatory vesicles^23,24^. Gene–environment interactions in this process further amplify this risk. Genome-wide association studies have found that individuals carrying FAM13A have an increased risk of developing COPD when exposed to PM2.5 compared to wild-type populations^25,26^. This suggests that traditional risk models may underestimate the contribution of gene–environment interactions, and that it is necessary to revisit prevention and control strategies from ‘single exposure control’ to ‘multicomponent integrated assessment’. In addition, the ‘hidden burden’ of occupational exposure is often overlooked. About 26.97% of COPD deaths globally are related to environmental/occupational risks. However, enterprises in low- and middle-income countries frequently exhibit inadequate provision of standardized protective equipment to workers, and there is a significant lag in the recognition of occupational diseases. This calls for the development of public health policies that take occupational exposures and genetic susceptibility into account in order to more effectively prevent and control the onset of COPD.

### Gender and age: neglected dimensions of difference

COPD exhibited significant gender differences that warranted study and attention. In general, men have a higher mortality rate from COPD, but females have a significantly increased susceptibility to certain specific environmental exposure conditions, especially long-term exposure to smoke from kitchen biomass fuels. This exposure not only raises females’ risk of developing COPD, but also results in a pathological phenotype that is predominantly characterized by small airway lesions, making them more likely to experience frequent acute exacerbations^27^. Research suggests that female vulnerability to specific environments may be related to differences in physiology, hormone levels, and immune responses^28^. However, few clinical trials have conducted gender-stratified analyses, which seriously overlooks the specific risks of female patients. In addition, data show that females have a higher error rate than males when using inhalation devices^29^, reflecting the lack of gender-sensitive medical strategies in COPD management. Aging is another challenge. This research indicated that individuals who are aged ≥60 years represent 92.90% of global fatalities caused by COPD. The distribution of resources dedicated to geriatric care is markedly unbalanced, which has a direct impact on both the health outcomes and overall quality of life experienced by elderly patients. This disparity in resource allocation can lead to inadequate care for many seniors, ultimately resulting in poorer health status and diminished well-being. In Japan, the integration of community-based rehabilitation centers and telemonitoring systems has significantly reduced acute exacerbation hospitalization rates among elderly patients. In contrast, the situation in rural India is relatively dire. Only 14% of health posts are equipped with basic lung function equipment, and the prevalence of home oxygen therapy is even less than 5%^30^. The lack of resources contributes to high mortality rates among elderly patients, reflecting a serious healthcare demand and service supply divide. In low- and middle-income countries, the early onset of aging due to lagging economic growth will exacerbate the pressure on existing healthcare systems, affecting the accessibility and quality of care.

### Future directions

The ASMR for COPD will continue to decline by 2051 and is projected to approach 19.97. This trend could be influenced by ongoing advancements in medical treatments, technology, public health policies (like smoking bans), and increased emphasis on early diagnosis and preventive care. While the forecast shows a potential decline, caution must be exercised in interpreting these projections. Predictive models are built on certain assumptions, including the effectiveness of public health interventions, demographic changes, and healthcare access. These factors may significantly sway outcomes. For instance, populations with increased air pollution or higher smoking prevalence may not see the same improvements. The COVID-19 pandemic has also impacted healthcare systems and may have indirectly affected COPD patients, either through exacerbations of their conditions or by changing the way healthcare is administered. The interactions between pandemic-related factors and COPD mortality projections are complex and warrant careful consideration. The reduction is promising, but being vigilant and proactive in a public health approach is critical. Mortality trends should be continuously monitored, risk factors reassessed, and strategies adjusted accordingly. This monitoring will enable timely responses to emerging trends or challenges in COPD management. The future of COPD management will rely heavily on sustained interventions, innovative healthcare strategies, and continued public awareness and education. By addressing the multiple factors that contribute to COPD mortality, we can strive for better health outcomes for affected populations in the future.

### Limitations

While this research provides valuable data and analysis, it also has some limitations. The study relies heavily on data from GBD 2021, which is extensive and authoritative, but may be affected by biases in data reporting, collection, and processing. Data quality varies significantly across countries and regions. Some low-income countries lack complete death registration and cause of death determination systems, and the data estimated by models may be biased. This data heterogeneity may affect the validity of global and interregional comparisons. Moreover, this study was mainly based on ecological data at the population level and could not infer causality at the individual level. The investigation primarily relied on correlation analysis rather than employing causal inference techniques. This choice presents challenges in accurately determining the direct causal connections between various risk factors and mortality rates. As a result, while associations may be identified, the study falls short of establishing definitive cause-and-effect relationships, which are crucial for a comprehensive understanding of how these risk factors may influence mortality outcomes. Residual confounding may affect the accuracy of attribution analysis of risk factors. Although GBD studies have adopted a systematic approach to risk factor attribution, due to the lack of individuallevel data, it is still difficult to fully control potential confounding factors such as occupational exposure intensity, differences in indoor air quality, and genetic susceptibility. Third, the overlap in clinical diagnosis between COPD and other respiratory diseases (e.g. asthma, bronchiectasis, pulmonary fibrosis), especially in low-resource areas, may lead to misdiagnosis or missed diagnosis due to limited diagnostic capacity. This taxonomic ambiguity may affect the accuracy of attribution of death and, in turn, mortality estimates. Underreporting of smoking exposure is widespread in multiple regions, particularly among women and adolescents. Due to socio-cultural factors or the stigma of smoking behavior, some people may conceal their smoking history in the survey, which leads to the underestimation of tobacco-related death burden. The rise of e-cigarettes and other newer tobacco products may create new exposure risks in younger populations that are not adequately captured in this study. Because e-cigarette use data are not well collected systematically worldwide, their potential impact on COPD mortality burden may be underestimated. A more comprehensive analysis would require integrating additional aspects and dimensions related to the disease, rather than solely focusing on mortality and its determinants. Finally, the uncertainty of the prediction model cannot be ignored. Although the ARIMA model makes predictions based on historical trends, it assumes that conditions such as public health intervention, policy implementation intensity, and medical access will remain unchanged in the future. Indeed, these externalities could change substantially as a result of policy changes, technological innovations, or public health emergencies (e.g. COVID-19) to affect actual future trends in COPD mortality. Future research should further expand data sources, improve statistical methods, explore in depth the complex mechanism of action of risk factors, and take into full consideration the multidimensional characteristics of cross-national inequalities in order to propose more targeted and feasible public health policy recommendations.

## CONCLUSIONS

This study delineates multifaceted trends and determinants shaping COPD mortality across global, regional, and national scales. While advancements in certain regions underscore the potential of targeted interventions, the persistent global burden of COPD highlights enduring systemic challenges. In the future, we need to continue to strengthen COPD prevention and control efforts, especially to develop differentiated prevention and control strategies for low- and lowmiddle SDI regions and high-risk populations. In addition, we need to pay further attention to COPD health inequalities and work to reduce the gaps in the response to COPD in different countries and regions.

## Supplementary Material



## Data Availability

Data used for the current study are publicly available through the GBD 2021 (http://ghdx.healthdata.org). Access to the data and codes used or analyzed in this research can be obtained from the corresponding author upon request.
