# Supplementary material for: Trends, cross-country inequalities and risk factors in the global burden of chronic obstructive pulmonary disease mortality, 1990–2021: a secondary data analysis of the Global Burden of Disease Study 2021
Source: Tob Induc Dis. 2026 Oct 16;24:10.18332/tid/228906. doi: 10.18332/tid/228906 (PMC13678534; doi:10.18332/tid/228906)
Supplement: Supplementary file 1 [file TID-24-188-s1.pdf]

Tobacco Induced Diseases

Supplementary file

© 2026 Sun Y. et al.

DOI:
10.18332/tid/228906

The content has been provided by the author(s) and has not been reviewed, verified, or endorsed by European Publishing. It may not have undergone peer review. The views, opinions, and recommendations expressed are solely those of the author(s) and do not necessarily reflect the position of European Publishing. European Publishing accepts no responsibility or liability for any consequences arising from the use of, or reliance on, this content.

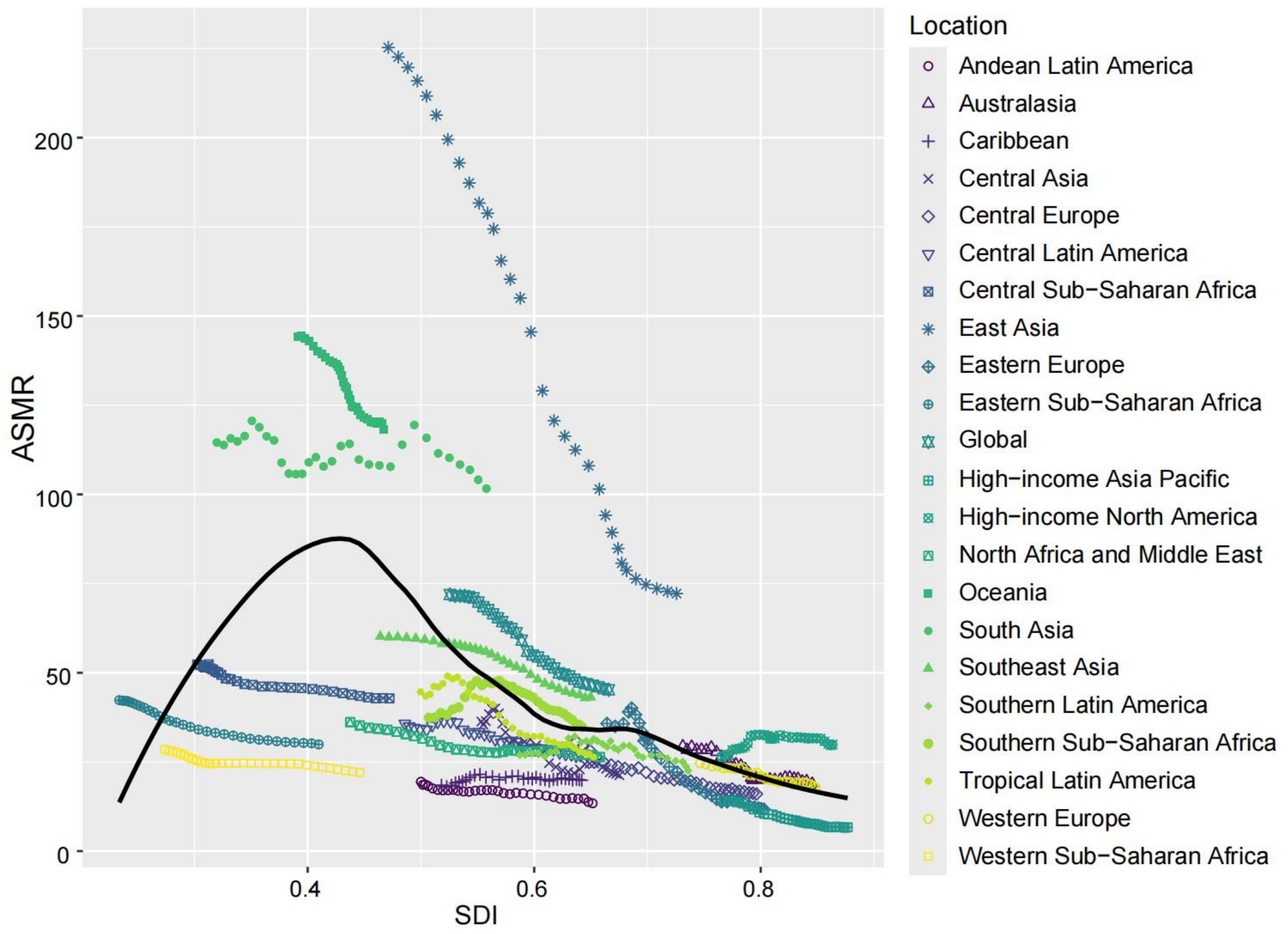

Figure S1. ASMR for COPD for 21 GBD regions by Socio-demographic Index, 1990–2021. Expected values based on Socio-demographic Index and disease rates in all locations are shown as the black line. ASMR, age-standardized mortality rate; EAPC, estimated annual percentage change; COPD, chronic obstructive pulmonary disease; GBD, Global Burden of Diseases, Injuries, and Risk Factors Study.

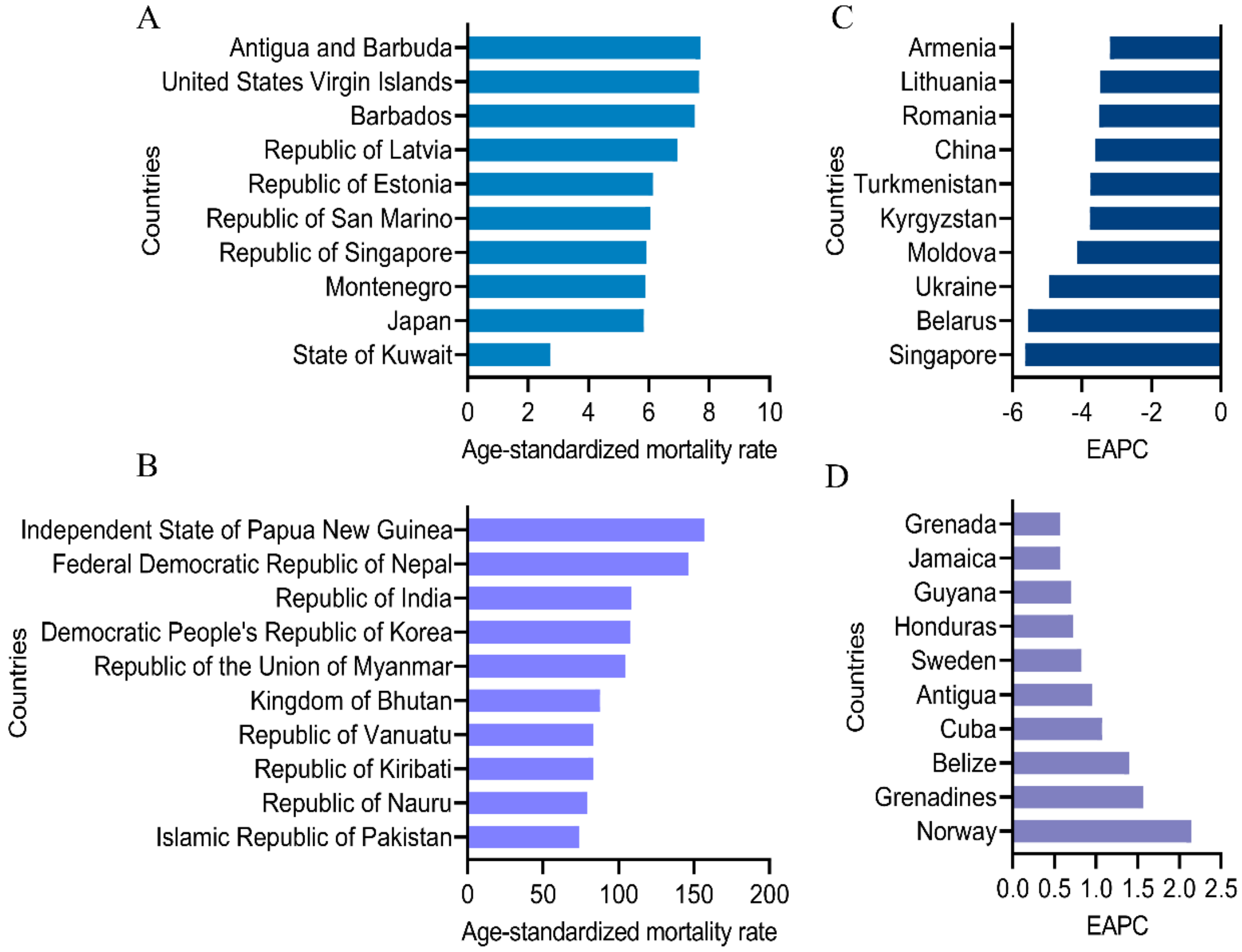

Figure S2. Ranking of countries with the highest and lowest ASMR and EAPC. (A) The top 10 countries with the lowest ASMR in 2021. (B) The top 10 countries with the highest ASMR in 2021. (C) The top 10 countries with the most pronounced decreases in EAPC from 1990 to 2021. (D) The top 10 countries with the most pronounced increases in EAPC from 1990 to 2021.

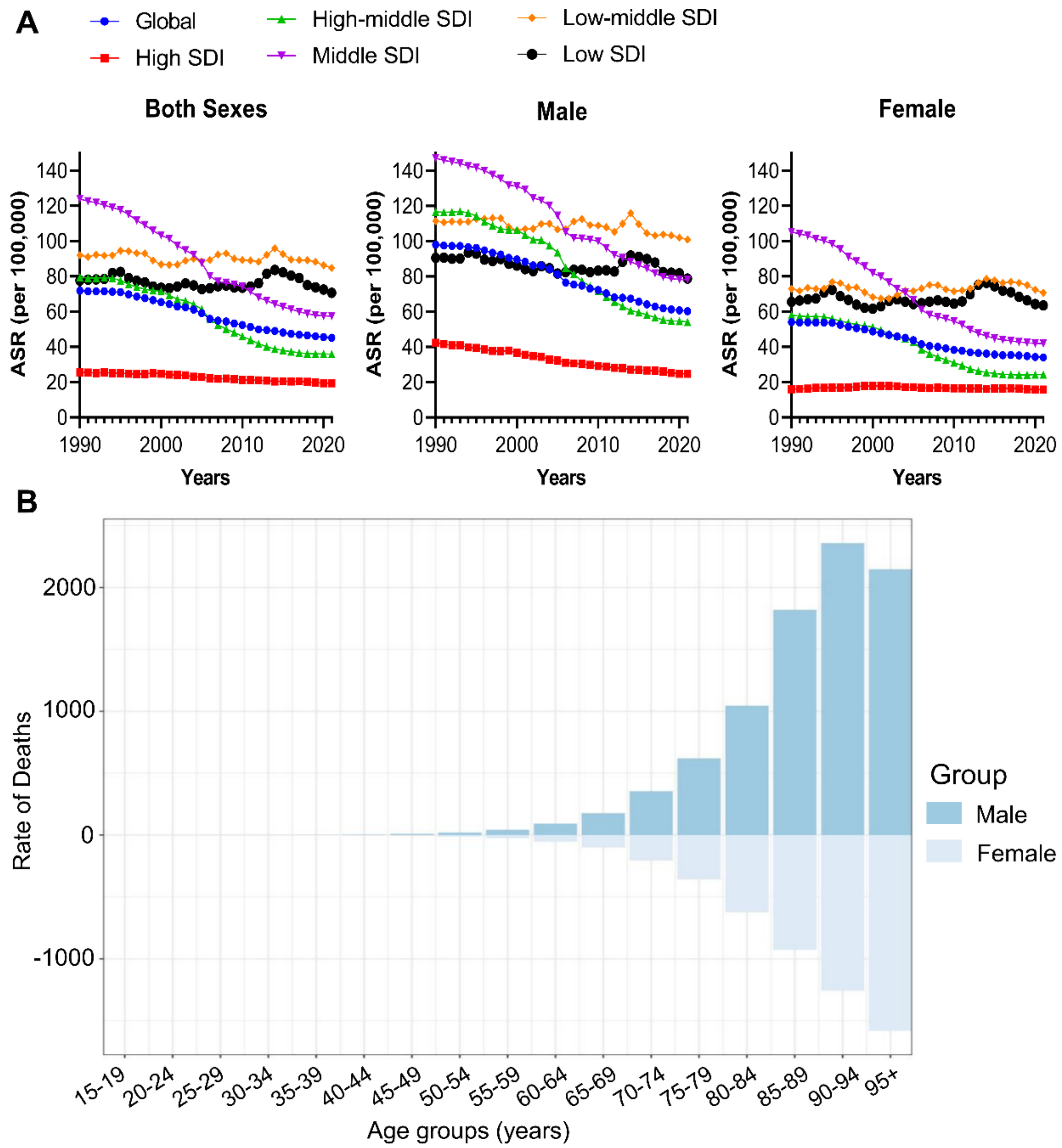

Figure S3. The influence of gender and age on mortality from COPD. (A) ASMR for COPD from 1990 to 2021. It is broken down by gender (all sexes, men and women) and SDI categories. (B) Global differences in COPD mortality by age group and sex in 2021. Negative values for females are used for visual contrast; all mortality rates are positive. Data source: Global Burden of Disease Study 2021.

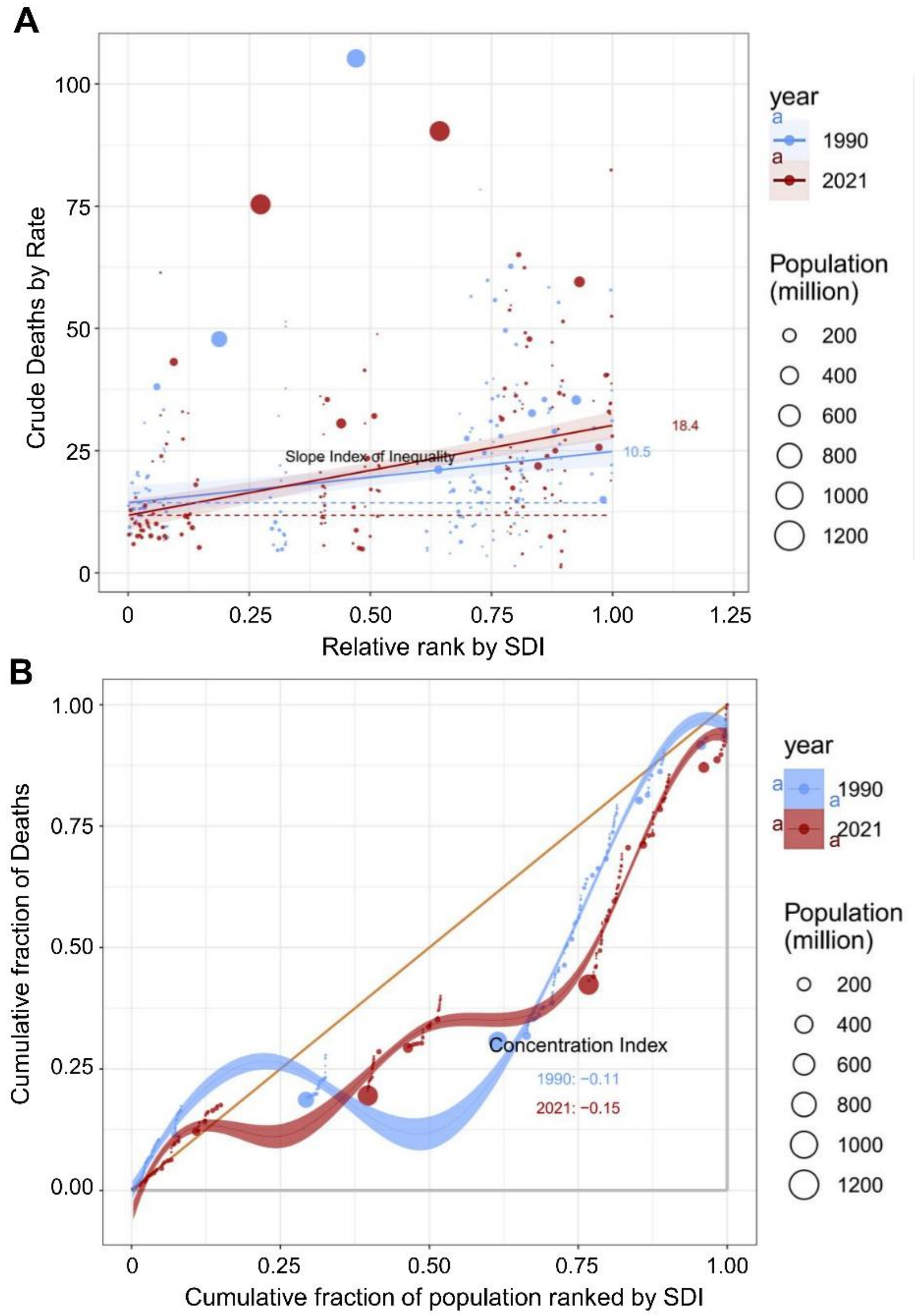

Figure S4. Health inequality analysis related to global COPD mortality in 1990 and 2021. (A) Slope Index of Inequality for global COPD mortality. Vertical axis: Age-standardized COPD mortality rate (deaths per 100 000 population). Horizontal axis: National Socio-demographic Index (SDI) ranked from 0 (lowest SDI) to 1 (highest SDI). The positive slopes indicate that lower-SDI countries have higher COPD mortality; the steeper 2021 slope shows widening absolute inequality. (B) Concentration Index curves for global COPD deaths. Vertical axis: Cumulative share of total COPD deaths (%). Horizontal axis: Cumulative share of world population ranked by SDI, from poorest (left) to richest (right). Thin grey diagonal: Line of equality (45°); if deaths were distributed proportionally to population the CI curve would coincide with this line. Curves lying below the diagonal indicate disproportionate mortality in

lower-SDI populations. The larger area between the curve and the diagonal denotes increased relative inequality. Data source: Global Burden of Disease Study 2021.

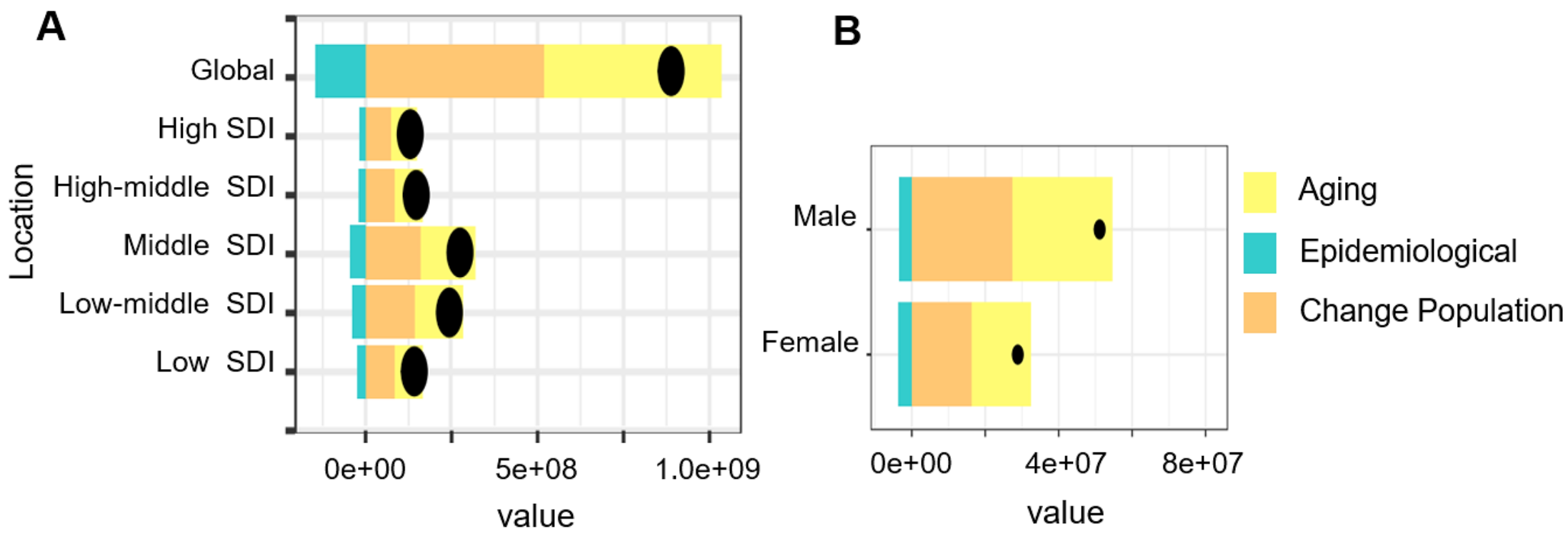

Figure S5. Decomposition analysis related to global COPD mortality. (A) Global and regional data on COPD in different SDI categories were compared from 1990 to 2021. (B) The contribution of different factors (aging, epidemiological change, and population change) to the overall burden of COPD was broken down and differentiated by sex. The X-axis displays the change in COPD-related deaths attributable to each contributing factor from 1990 to 2021. Black dots indicate the overall change in contributions across all three components. For each individual component, a positive value reflects an increase in the number of COPD deaths associated with that factor, whereas a negative value signifies a reduction in the number of deaths attributable to that specific component. Data source: Global Burden of Disease Study 2021.

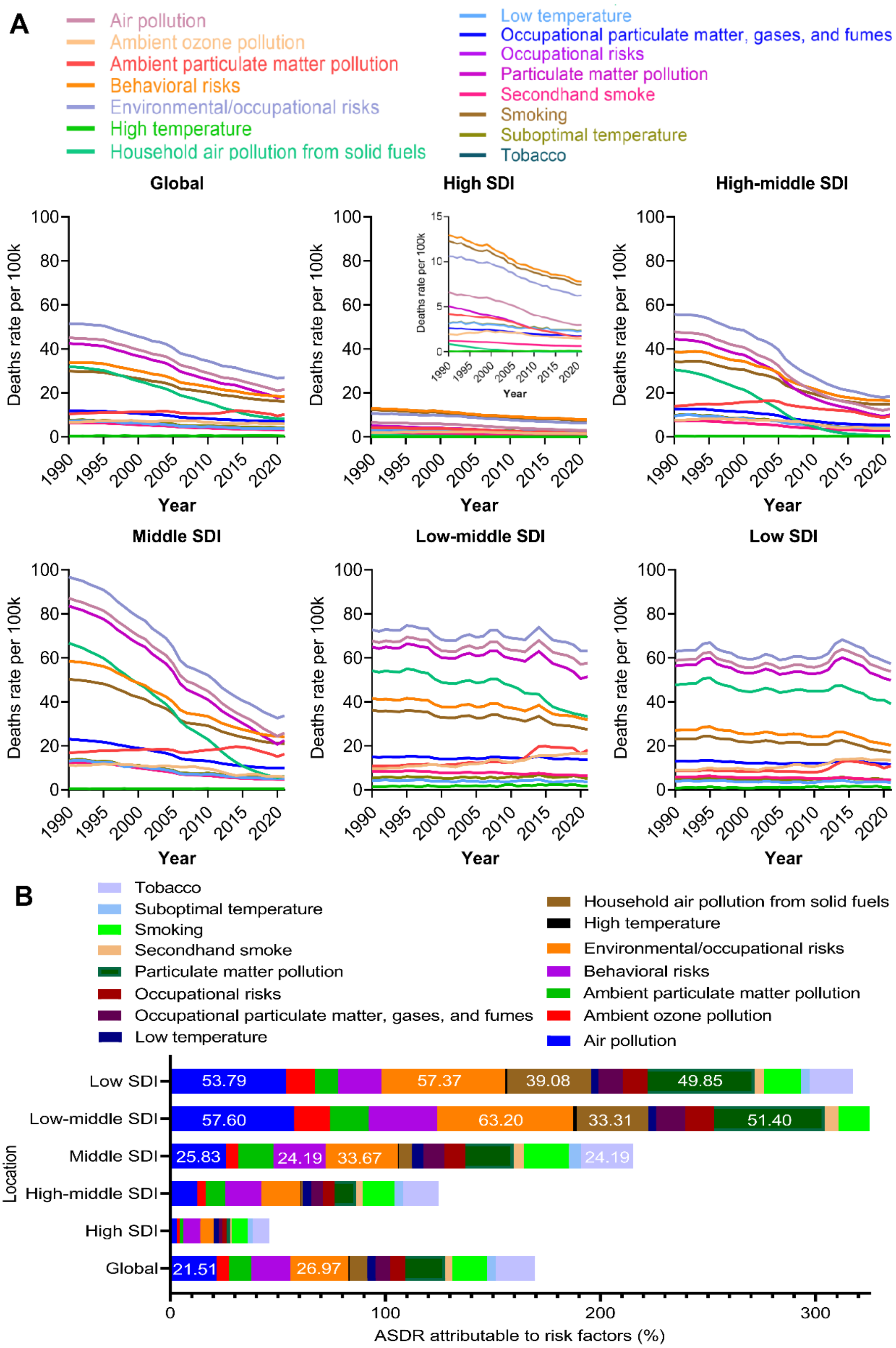

Figure S6. Analysis of COPD mortality trends and risk factor distribution across SDI regions. (A) The evolution of COPD mortality trends attributable to major risk factors across regions categorized by SDI from 1990 to 2021. (B) Distribution of COPD mortality due to risk factors in different SDI categories in 2021. Data source: Global Burden of Disease Study 2021.

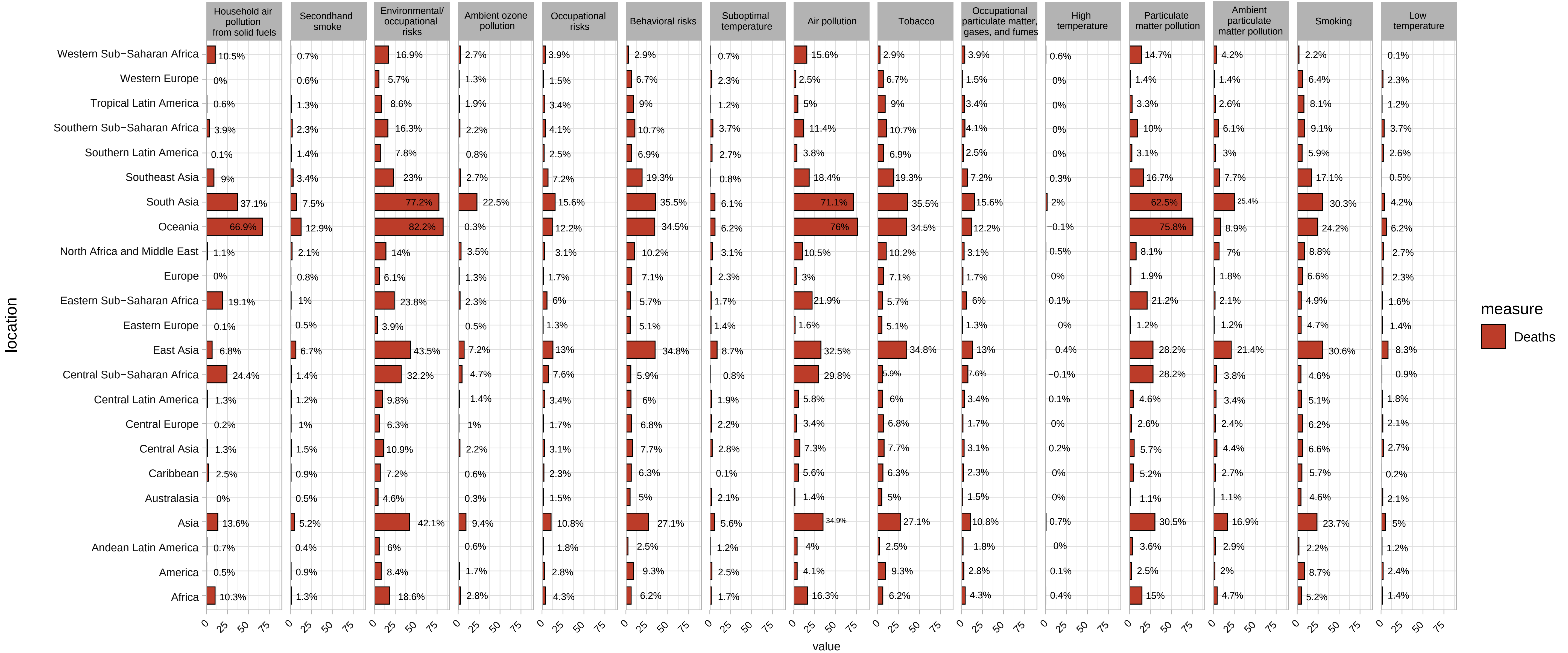
Household air pollution from solid fuels
Secondhand smoke
Environmental/ occupational risks
Ambient ozone pollution
Occupational risks
Behavioral risks
Suboptimal temperature
Air pollution
Tobacco
Occupational particulate matter, gases, and fumes
High temperature
Particulate matter pollution
Ambient particulate matter pollution
Smoking
Low temperature
Western Sub−Saharan Africa
Western Europe
Tropical Latin America
Southern Sub−Saharan Africa
Southern Latin America
Southeast Asia
South Asia
Oceania
North Africa and Middle East
Europe
Eastern Sub−Saharan Africa
Eastern Europe
East Asia
Central Sub−Saharan Africa
Central Latin America
Central Europe
Central Asia
Caribbean
Australasia
Asia
Andean Latin America
America
Africa
10.5% 0% 0.6% 3.9% 0.1% 9% 37.1% 66.9% 1.1% 0% 19.1% 0.1% 6.8% 24.4% 1.3% 0.2% 1.3% 2.5% 0% 13.6% 0.7% 0.5% 10.3%
0.7% 0.6% 1.3% 2.3% 1.4% 3.4% 7.5% 12.9% 2.1% 0.8% 1% 0.5% 6.7% 1.4% 1.2% 1% 1.5% 0.9% 0.5% 5.2% 0.4% 0.9% 1.3%
16.9% 5.7% 8.6% 16.3% 7.8% 23% 77.2% 82.2% 14% 6.1% 23.8% 3.9% 43.5% 32.2% 9.8% 6.3% 10.9% 7.2% 4.6% 42.1% 6% 8.4% 18.6%
2.7% 1.3% 1.9% 2.2% 0.8% 2.7% 22.5% 0.3% 3.5% 1.3% 2.3% 0.5% 7.2% 4.7% 1.4% 1% 2.2% 0.6% 0.3% 9.4% 0.6% 1.7% 2.8%
3.9% 1.5% 3.4% 4.1% 2.5% 7.2% 15.6% 12.2% 3.1% 1.7% 6% 1.3% 13% 7.6% 3.4% 1.7% 3.1% 2.3% 1.5% 10.8% 1.8% 2.8% 4.3%
2.9% 6.7% 9% 10.7% 6.9% 19.3% 35.5% 34.5% 10.2% 7.1% 5.7% 5.1% 34.8% 5.9% 6% 6.8% 7.7% 6.3% 5% 27.1% 2.5% 9.3% 6.2%
0.7% 2.3% 1.2% 3.7% 2.7% 0.8% 6.1% 6.2% 3.1% 2.3% 1.7% 1.4% 8.7% 0.8% 1.9% 2.2% 2.8% 0.1% 2.1% 5.6% 1.2% 2.5% 1.7%
15.6% 2.5% 5% 11.4% 3.8% 18.4% 71.1% 76% 10.5% 3% 21.9% 1.6% 32.5% 29.8% 5.8% 3.4% 7.3% 5.6% 1.4% 34.9% 4% 4.1% 16.3%
2.9% 6.7% 9% 10.7% 6.9% 19.3% 35.5% 34.5% 10.2% 7.1% 5.7% 5.1% 34.8% 5.9% 6% 6.8% 7.7% 6.3% 5% 27.1% 2.5% 9.3% 6.2%
3.9% 1.5% 3.4% 4.1% 2.5% 7.2% 15.6% 12.2% 3.1% 1.7% 6% 1.3% 13% 7.6% 3.4% 1.7% 3.1% 2.3% 1.5% 10.8% 1.8% 2.8% 4.3%
0.6% 0% 0% 0% 0% 0.3% 2% −0.1% 0.5% 0% 0.1% 0% 0.4% −0.1% 0.1% 0% 0.2% 0% 0% 0.7% 0% 0.1% 0.4%
14.7% 1.4% 3.3% 10% 3.1% 16.7% 62.5% 75.8% 8.1% 1.9% 21.2% 1.2% 28.2% 28.2% 4.6% 2.6% 5.7% 5.2% 1.1% 30.5% 3.6% 2.5% 15%
4.2% 1.4% 2.6% 6.1% 3% 7.7% 25.4% 8.9% 7% 1.8% 2.1% 1.2% 21.4% 3.8% 3.4% 2.4% 4.4% 2.7% 1.1% 16.9% 2.9% 2% 4.7%
2.2% 6.4% 8.1% 9.1% 5.9% 17.1% 30.3% 24.2% 8.8% 6.6% 4.9% 4.7% 30.6% 4.6% 5.1% 6.2% 6.6% 5.7% 4.6% 23.7% 2.2% 8.7% 5.2%
0.1% 2.3% 1.2% 3.7% 2.6% 0.5% 4.2% 6.2% 2.7% 2.3% 1.6% 1.4% 8.3% 0.9% 1.8% 2.1% 2.7% 0.2% 2.1% 5% 1.2% 2.4% 1.4%
0 25 50 75
location
value
measure
Deaths

Figure S7. The influence of risk factors on COPD mortality in different regions in 2021. Data source: Global Burden of Disease Study 2021.

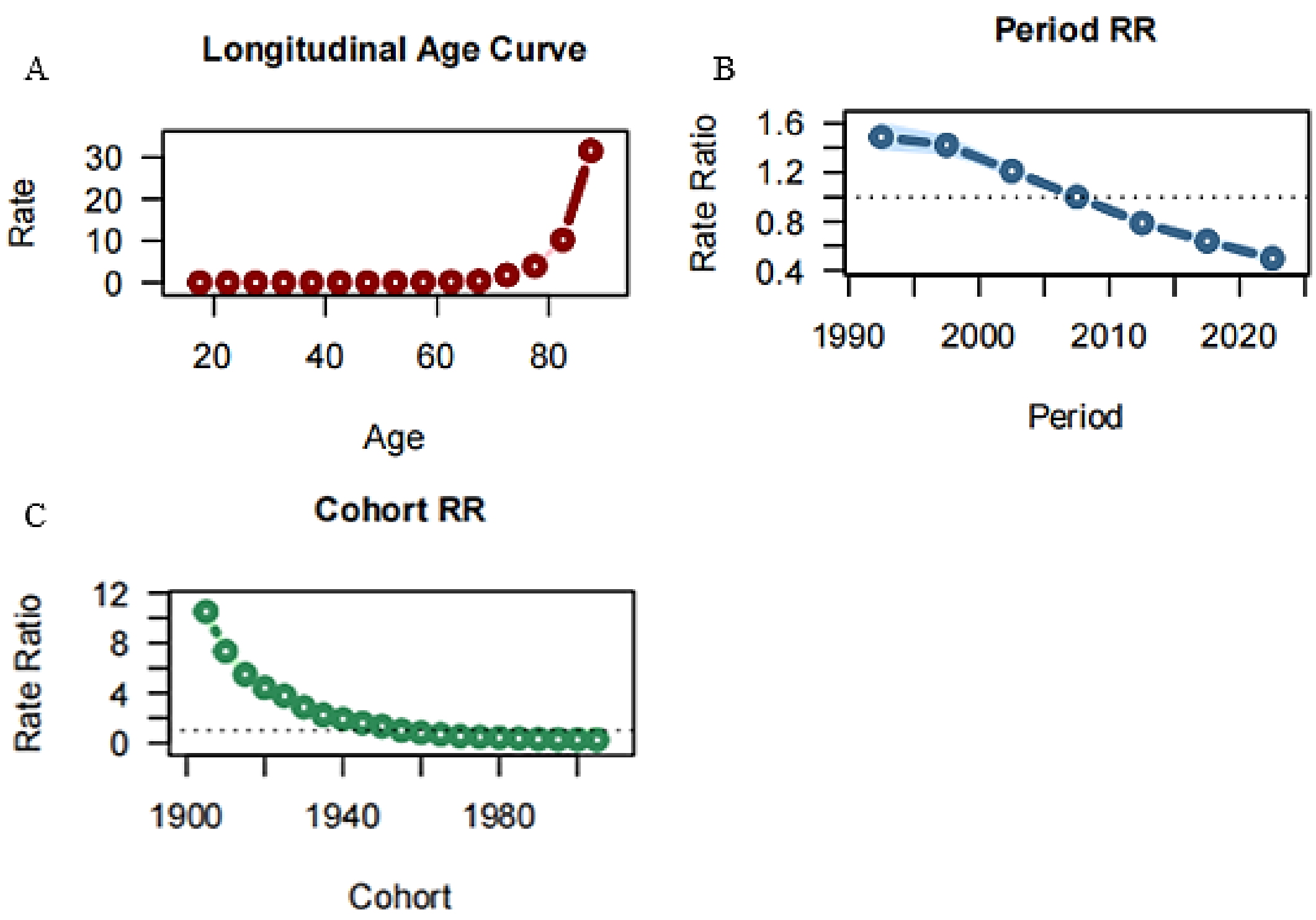

Figure S8. Age-period-cohort effect on ASMR of COPD. (A) Age-specific COPD mortality rate. (B) Period-based COPD mortality rate. A Rate Ratio of more than 1 indicates a higher relative risk of death/incidence compared with the reference period, while a Rate Ratio of less than 1 indicates a lower relative risk of death/incidence compared with the reference period. (C) Cohort-based COPD mortality rate. A Rate Ratio greater than 1 indicates a higher relative risk of death/incidence compared with the reference cohort, while a Rate Ratio less than 1 indicates a lower relative risk of death/incidence compared with the reference cohort. ASMR, age-standardized mortality rate; COPD, chronic obstructive pulmonary disease.
